# Mechanisms of bradycardia in premature infants: Aerodigestive–cardiac regulatory–rhythm interactions

**DOI:** 10.14814/phy2.14495

**Published:** 2020-07-08

**Authors:** Kathryn A. Hasenstab‐Kenney, Jenny Bellodas Sanchez, Varsha Prabhakar, Ivan M. Lang, Reza Shaker, Sudarshan R. Jadcherla

**Affiliations:** ^1^ Innovative Neonatal and Infant Feeding Disorders Research Program Center for Perinatal Research The Research Institute at Nationwide Children's Hospital Columbus OH USA; ^2^ Division of Neonatology Pediatric Gastroenterology and Nutrition Nationwide Children's Hospital Columbus OH USA; ^3^ MCW Dysphagia Institute Division of Gastroenterology and Hepatology Department of Medicine Medical College of Wisconsin Milwaukee WI USA; ^4^ Department of Pediatrics The Ohio State University College of Medicine Columbus OH USA

**Keywords:** apnea, bradycardia, cardiorespiratory and life‐threatening events, pharyngoesophageal manometry, swallowing

## Abstract

**Objective:**

Eating difficulties coupled with cardiorespiratory spells delay acquisition of feeding milestones in convalescing neonates, and the mechanisms are unclear. Aims were to examine and compare the pharyngoesophageal–cardiorespiratory (PECR) response characteristics: (a) in control neonates and those with recurrent bradycardia spells; and (b) during pharyngeal stimulation when bradycardia occurs versus when no bradycardia occurs.

**Methods:**

Preterm infants (*N* = 40, 27 ± 3 weeks gestation), underwent concurrent pharyngoesophageal manometry, electrocardiography, respiratory inductance plethysmography, and nasal airflow thermistor to evaluate pharyngoesophageal motility, heart rate (HR), and respiration during graded abrupt pharyngeal sterile water stimuli. Infants with recurrent bradycardia (*N* = 28) and controls (*N* = 12) were evaluated at 38 (38–40) and 39 (38–40) weeks postmenstrual age, respectively. Comparisons were performed (a) between study and control groups; and (b) among HR responses of <80 BPM, 80–100 BPM, and >100 BPM.

**Results:**

Overall, characteristics of PECR responses in infants with a history of recurrent bradycardia (vs. controls) did not differ (*p* > .05). However, when pharyngeal stimulus induced severe bradycardia (<80 BPM): prolonged respiratory rhythm change, increased pharyngeal activity, increased esophageal dysmotility (as evidenced by prolonged esophageal inhibition and motor activity), and prolonged lower esophageal sphincter relaxation were noted (all *p* < .05).

**Conclusions:**

In control infants and those with recurrent bradycardia, pharyngeal stimulation results in similar PECR response characteristics. However, when severe bradycardia occurs, PECR response characteristics are distinct. The mechanisms of severe bradycardia spells are related to abnormal prolongation of vagal inhibitory effects on cardiorespiratory rhythms in conjunction with prolonged esophageal inhibition and delays with terminal swallow.

## Graphical abstract

Pharyngeal stimulation shows that pharyngeal‐esophageal‐cardiac‐respiratory (PECR) response characteristics are not different in infants with recurrent bradycardia. However, PECR dysfunction occurs during severe bradycardia and its mechanisms are related to abnormal prolongation of vagal inhibitory effects on cardiac‐respiratory rhythms in conjunction with prolonged esophageal inhibition and delays with terminal swallow.

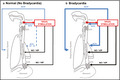

## 1 INTRODUCTION

Cardiorespiratory dysregulation and swallowing difficulties are frequent concerns in convalescing premature infants interfering with timely hospital discharge. Discharge criteria by the American Academy of Pediatrics for preterm infants include physiological maturity with stable cardiorespiratory function, and exhibition of competent feeding without cardiorespiratory compromise (American Academy of Pediatrics Committee on Fetus & Newborn, 2008). Although objective evidence is lacking, it is common practice to delay discharge by a 5‐consecutive day free of any cardiorespiratory events so as to prevent sudden unexplained life‐threatening events after discharge, as such occurrence may result in re‐hospitalization (Jefferies, Canadian Paediatric Society, & Fetus and Newborn Committee, 2014). The prevalence of cardiorespiratory events in convalescing premature infants varies widely but can be approximated at 12.5%. These events frequently resolve by 36 weeks postmenstrual age (PMA), but may still be present up to and beyond 44 weeks PMA, costing an additional $4,400–$35,000 per infant (Bakewell‐Sachs, Medoff‐Cooper, Escobar, Silber, & Lorch, 2009; Eichenwald, Aina, & Stark, 1997; Montenegro et al., 2017).

Cardiorespiratory events such as apnea and bradycardia may occur spontaneously, during feeding, and rarely may be accompanied by gastroesophageal reflux (Chandrasekharan, Rawat, Reynolds, Phillips, & Lakshminrusimha, 2018; Eichenwald, 2016; Henderson‐Smart, Butcher‐ Puech, & Edwards, 1986). The Neonatal Resuscitation Program (NRP) defines bradycardia as heart rate (HR) less than 100 beats per minute (bpm) for neonates (American Academy of Pediatrics & American Heart Association, 2016); and recommends intervention for HR < 100 bpm. In contrast, the Pediatric Advanced Life Support provider manual defines it as HR less than 80 bpm in sleeping state for infants younger than 3 months of age (Chameides et al., 2011). The NRP guidelines for intervention based on HR are mainly designed for active resuscitation in the delivery room; which makes it difficult to extrapolate these recommendations to older neonatal intensive care unit (NICU) infants suffering from bradycardic/apneic events. The etiology of which are multifactorial and remains to be systematically studied. A vast variety of conditions could lead to apnea and bradycardia in infants; which makes management and etiological diagnosis challenging to clinicians. Abnormalities during swallowing are increasingly being implicated as a trigger for cardiorespiratory events (Davies, Koenig, & Thach, 1989; Duncan, Amirault, Mitchell, Larson, & Rosen, 2017; Hasenstab & Jadcherla, 2014; Thach, 2001); however, the exact mechanism remains poorly understood. Our previous study in 48 preterm infants, showed abnormal PECR responses with HR decrease (Hasenstab, Nawaz, Lang, Shaker, & Jadcherla, 2019); which raised our interest in further evaluation of aerodigestive reflexes in preterm infants with bradycardia concerns. Although upper airway‐digestive reflexes such as apnea, swallowing, cough, laryngeal chemoreflex (upstream reflexes) have been undertaken before (Davies, Koenig, & Thach, 1985; Davies et al., 1989; Pickens, Schefft, & Thach, 1988), there is limited knowledge and investigations about esophageal motility or lower esophageal sphincter (LES) function (downstream reflexes) (Hasenstab & Jadcherla, 2014; Hasenstab et al., 2019; Hasenstab, Sitaram, Lang, Shaker, & Jadcherla, 2018; Jadcherla, Gupta, Stoner, Fernandez, & Shaker, 2007; Jadcherla et al., 2016; Jadcherla, Shubert, et al., 2015) or understanding of these concepts for improved diagnostic and focused management strategies. Therefore, we investigated the pharyngoesophageal and cardiorespiratory responses to assess cross‐system functions of swallowing in convalescing preterm‐born neonates with bradycardia concerns.

Our study aims were to compare pharyngoesophageal–cardiorespiratory (PECR) response characteristics in preterm NICU infants: (a) with recurrent bradycardia versus controls; and (b) during responses resulting in severe bradycardia (HR < 80 BPM) and bradycardia (HR 80–100 BPM) versus no bradycardia (HR > 100 BPM). We hypothesized that PECR characteristics are: (a) distinct in NICU infants with history of recurrent bradycardia; and (b) specific inter‐regulatory mechanisms between cross‐systems are dysfunctional during bradycardic events.

## 2 MATERIALS AND METHODS

### 2.1 Subjects

Data were examined from 40 preterm born NICU infants (23 male) born at 27.0 ± 3.1 weeks gestational age who underwent concurrent cardiorespiratory and pharyngo‐esophageal motility protocols. All studies were performed at the Infant Feeding Disorders Innovative Research Program at Nationwide Children's Hospital, Columbus, OH, USA. Data from infants were included for analysis if they met the following criteria: (a) born preterm (gestational age < 37 weeks); (b) referred for feeding/swallowing evaluation; (c) near full‐term status (between 36–40 weeks PMA) and enterally feeding at time of evaluation; (d) were discharged orally feeding. Infants were excluded if they had known chromosomal and genetic disorders or airway‐digestive tract surgery. Prior to the study, informed parental consent and Institutional Research Review Board approval at Nationwide Children's Hospital were obtained. Health Insurance Portability and Accountability policy guidelines were followed. Subject safety was continuously monitored by the study physician and registered nurse at the bedside during the procedure.

### 2.2 Experimental protocol

As previously published, infants underwent concurrent water perfusion manometry with pharyngeal provocation, and cardiorespiratory monitoring to test pharyngoesophageal motility, HR, and respiratory responses (Hasenstab et al., 2018, 2019; Jadcherla et al., 2007; Jadcherla et al., 2009; Jadcherla et al., 2016; Jadcherla, Shubert, et al., 2015). Briefly, a custom designed silicone catheter (Dentsleeve International, Mui Scientific) with pressure channels (pharynx, proximal‐, middle‐, and distal‐esophagus, stomach, and pharyngeal infusion port for infusions) and pressure sleeves (upper and LES) attached to a water perfusion manometry system with amplifiers and transducers (Solar GI; Laborie Medical Technologies) to record pharyngoesophageal motility in response to pharyngeal stimulus was placed by the study physician nasally using the pull‐through technique in the supine lying infant. Catheter selection was dependent on infant weight at time of evaluation. (Hasenstab et al., 2018, 2019; Jadcherla et al., 2007, 2009, 2016; Jadcherla, Shubert, et al., 2015). Prior to catheter placement, calibration occurred at the level of the subject's esophagus (Gupta & Jadcherla, 2006). After catheter placement, the infant was given about 10–15 min for adaptation prior to pharyngeal infusion protocol initiation. Infusion protocol included graded volumes (0.1, 0.3, and 0.5 ml in triplicate) of sterile water administered via the pharyngeal infusion port by the study physician (Hasenstab et al., 2018, 2019; Jadcherla et al., 2007, 2009, 2016; Jadcherla, Shubert, et al., 2015). Concurrently, dual‐band respiratory inductance plethysmography (Respitrace; Viasys), nasal thermistor (Integra Life Sciences), and electrocardiogram (Laborie Medical Technologies or BioRadio 150 Great Lakes NeuroTechnologies) were utilized to evaluate cardiorespiratory rhythms (Hasenstab et al., 2018, 2019; Jadcherla et al., 2007, 2009, 2016; Jadcherla, Shubert, et al., 2015). The subject was continuously monitored by the study physician and registered nurse throughout the study.

### 2.3 Data analysis

#### 2.3.1 Selection of participants with significant bradycardia characteristics

Patient charts were reviewed in the electronic health record system at Nationwide Children's Hospital (Epic; Epic Systems Corporation) for pertinent data characteristics during actual bradycardia events including: (a) bradycardia occurrences (#), rate (BPM), and duration (s); (b) desaturation (defined as <85% oxygen saturation) and its magnitude (% oxygen saturation); (c) intervention type (%) as performed by a medical provider, defined as mild (gentle tactile stimulation, repositioning, or pause in feeding), moderate (moderate stimulation ± repositioning or suctioning, or vigorous tactile stimulation only), severe (requiring chest compressions, bagging, blow by oxygen, vigorous tactile stimulation + suctioning, or FiO_2_ breaths), or none (infant self‐recovered); and (d) and occurrence type (%) defined as feeding related (during or shortly after feeds), gastrointestinal related (emesis or stooling), or spontaneous (neither feeding or gastrointestinal related).

#### 2.3.2 Data acquisition and analysis of pharyngoesophageal and cardiorespiratory characteristics

Data analysis was performed using MMS analysis software (v. 2.04; Laborie Medical Technologies) and VivoSense software (v 2.8; Vivonoetics) to evaluate pharyngoesophageal and cardiorespiratory changes to pharyngeal provocation as previously published (Hasenstab et al., 2018, 2019; Jadcherla et al., 2007, 2009, 2016; Jadcherla, Shubert, et al., 2015). Regional response latency (infusion onset to response onset, s) and response duration (response latency to response offset, s) were analyzed for each region including the pharynx, esophagus, LES, heart, and respiratory system (Hasenstab et al., 2018, 2019; Jadcherla et al., 2007, 2009, 2016; Jadcherla, Hasenstab, Shaker, & Castile, 2015; Jadcherla, Shubert, et al., 2015; Pena et al., 2010). Additional variables specific to each region included: (a)* pharynx* (Hasenstab et al., 2018, 2019; Jadcherla et al., 2007; Jadcherla, Shubert, et al., 2015)—recruitment (#) as the number of pharyngeal peaks per stimulus, frequency (Hz) as 1/duration of each peak to peak interval, variability (s) as the standard deviation of peak to peak intervals, stability (#) as standard deviation/mean of pharyngeal peak to peak intervals, and terminal swallow prevalence (%)—terminal swallow defined as the occurrence of final primary peristalsis in a swallowing sequence after which there is restoration of respiratory rhythm and digestive motility rhythms to normalcy; (b)* esophagus* (Hasenstab et al., 2018, 2019; Jadcherla, Hasenstab, et al., 2015; Jadcherla et al., 2016)—inhibition (s) as the time between pharyngeal response onset to esophageal response onset, and terminal swallow prevalence (%) as swallow initiating airway and digestive normalcy (reestablishment of esophageal baseline quiescence and baseline respiratory rhythm); (c)* LES* (Jadcherla, Shubert, et al., 2015; Pena et al., 2010)—basal tone (mmHg) as resting tone prior to infusion, relaxation prevalence (%) as presence of relaxation, nadir relaxation (mmHg) as lowest relaxation pressure; (d) *cardiac rates and rhythm* (Hasenstab et al., 2019)—basal HR as average resting HR prior to infusion, HR response prevalence (%) defined as a 10% change from resting baseline, HR response (BPM) as lowest HR, HR decrease magnitude (%) as percentage change from basal HR, basal R–R interval (s) as average beat to beat interval durations during resting baseline, and R–R response (s) as peak interval duration during HR response; (e)* respiratory system* (Hasenstab et al., 2019; Jadcherla, Hasenstab, et al., 2015; Jadcherla et al., 2016)—prevalence of respiratory rhythm change (%) as change from resting breathing rhythms, and prevalence of deglutition apnea (%) defined as cessation of nasal, chest, and abdominal breathing with pharyngeal swallowing; and (f) *symptom prevalence* (%) as presence of additional symptoms other than apnea/bradycardia/desaturation such as movement/arching, coughing, sneezing, etc (Collins, Hasenstab, Nawaz, & Jadcherla, 2019; Jadcherla, Hasenstab, et al., 2015).

### 2.4 Statistical analysis

Statistical analysis was performed using R (v 3.5.1; R Core Team, 2018). Nonparametric and chi‐squared tests were used to compare demographic characteristics between cases (infants with recurrent bradycardia) and controls (infants without recurrent bradycardic events). Linear mixed models and generalized estimating equations were used for PECR characteristics to compare (a) cases versus controls; and (b) bradycardic rhythm severity (HR < 80 BPM vs. HR 80–100 vs. HR > 100 BPM). Note: data from case and control infants were combined for these bradycardic rhythm severity comparisons. All statistical models were controlled for PMA and infusion volume. *Benjamini–Hochberg* *p*‐value adjustment was used for multiple comparisons (Williams, Jones, & Tukey, 1999). Data are presented as median (interquartile range), mean ± *SEM*, %, or odds ratios (95% confidence intervals) unless otherwise noted.

## 3 RESULTS

### 3.1 Demographic and clinical characteristics

In infants with recurrent bradycardia (*N* = 28) versus control (*N* = 12), respectively, characteristics are as follows: (a) *at birth*—gestational age was 26.5 (24.1–29.4) versus 25.9 (24.3–27.7) weeks, *p* = .6, weight was 0.9 (0.7–1.3) versus 0.7 (0.7–1.2) kg, *p* = .4; (b) *at evaluation*—PMA was 38.4 (37.6–40.0) versus 39.3 (38.1–40.1) weeks, *p* = .1, weight was 2.6 (2.3–2.9) versus 2.7 (2.4–3.2) kg, *p* = .4; BPD prevalence was 68% versus 92%, *p* = .1; neurologic condition prevalence was 18% versus 25%, *p* = .2; and number of bradycardic events within 2 weeks of evaluation were 19 (7–46) versus 0 (0–1), *p* < .01; (c) *at discharge*—weight was 3.4 (2.8–4.3) versus 3.7 (3.4–4.3) kg, *p* = .4, and length of hospitalization was 114 (78–130) versus 117 (97–158) days, *p* = .2.

Further description of the nature of bradycardia event history via electronic health records, collected 2 weeks prior to evaluation, in infants with recurrent bradycardia (*N* = 28) are as follows: (a) total number of bradycardia events were 540 for HR 68 (60–76) BPM for duration of 15 (10–27) s, and associated with oxygen desaturation in 393/537 (73%) with magnitude of 66 (56–74)%; (b) spontaneous events in 275/516 (53%), with feeds in 227/516 (44%), and with gastrointestinal events in 14/516 (3%); (c) severe intervention required in 81/517 (16%), moderate intervention in 127/517 (24%), mild intervention in 164/517 (32%), and self‐recovery in 145/517 (28%).

### 3.2 Pharyngeal stimuli induced PECR responses

#### 3.2.1 Subjects with recurrent bradycardia versus controls

Among subjects with recurrent bradycardia, the risk of actual bradycardia upon pharyngeal stimulation did not increase significantly, OR (95% CI) was 2.8 (0.5–14.9) for HR < 80 BPM, *p* = .2, and 1.6 (0.5–4.8) for HR 80–100 BPM, *p* = .4. Of the pharyngeal stimuli administered (*N* = 287), for infants with recurrent bradycardia versus control: (a) stimulus flow (ml/s)—as the rate at which fluid stimulus is given—was 0.27 ± 0.01 versus 0.28 ± 0.01, *p* = .4; and (b) threshold volume (ml)—as the least amount of fluid stimulus at which esophageal response begins, as evidence by water perfusion manometry tracings—was 0.18 ± 0.01 versus 0.20 ± 0.01, *p* = .2, respectively. PECR response characteristics were not significantly different between infants with recurrent bradycardia versus controls (Table 1).

**TABLE 1:** Comparison of pharyngeal stimulus induced pharyngoesophageal‐cardiorespiratory (PECR) responses between infants with recurrent bradycardia and controls

Characteristic	Recurrent bradycardia (*N* = 28 infants)	Control (*N* = 12 infants)	*p*‐value
Pharyngeal stimulus duration, s	9.1 ± 0.5	7.4 ± 0.8	.1
Pharyngeal phase			
Recruitment, # peaks/stimulus	6.4 ± 0.5	7.0 ± 0.8	.5
Response latency, s	4.2 ± 0.5	4.5 ± 0.8	.8
Response duration, s	17.0 ± 1.8	19.7 ± 2.9	.4
Frequency, Hz	0.63 ± 0.04	0.65 ± 0.06	.8
Peak–peak variability, s	2.2 ± 0.3	2.5 ± 0.5	.6
Stability, *SD*/ $\bar{X}$	0.7 ± 0.1	0.8 ± 0.1	.5
Esophageal phase			
Response latency, s	11.7 ± 0.8	10.0 ± 1.2	.3
Inhibition, s	8.4 ± 0.7	6.8 ± 1.0	.2
Response duration, s	17.5 ± 2.3	20.6 ± 3.6	.5
LES			
Basal tone, mmHg	17 ± 2	18 ± 2	.8
LESR response latency, s	5.7 ± 0.6	5.2 ± 0.9	.7
LESR onset to nadir, s	2.1 ± 0.1	2.2 ± 0.2	.5
LESR nadir duration, s	15.5 ± 1.3	17.3 ± 2.1	.5
LESR nadir pressure, mmHg	−0.1 ± 0.7	0.6 ± 1.1	.6
Cardiac			
Basal HR, bpm	151 ± 2	149 ± 4	.6
HR response, bpm	134 ± 4	138 ± 5	.6
HR response latency, s	11.7 ± 1.0	10.3 ± 1.6	.5
HR response duration, s	12.3 ± 1.9	14.5 ± 3.2	.6
HR decrease magnitude, %	32.4 ± 2.0	26.8 ± 3.2	.1
Basal R–R interval, s	0.40 ± 0.01	0.41 ± 0.01	.6
R–R response, s	0.48 ± 0.02	0.45 ± 0.02	.3
Respiratory			
Rhythm change latency, s	3.8 ± 0.4	3.9 ± 0.6	.9
Rhythm change duration, s	23.1 ± 2.6	25.7 ± 4.0	.6
Deglutition apnea duration, s	5.5 ± 0.6	4.6 ± 1.0	.5

Note

Data presented as Mean ± *SE* or as stated.

No significant differences were noted between infants with recurrent bradycardia and control.

Abbreviations: *SD*, standard deviation; $\bar{X}$, mean; LES, lower esophageal sphincter; LESR, lower esophageal sphincter relaxation; HR, heart rate; bpm, beats per minute.

#### 3.2.2 Responses resulting in Bradycardic Rhythms (HR: <80 vs. 80–100 vs. >100)

**FIGURE 1:**
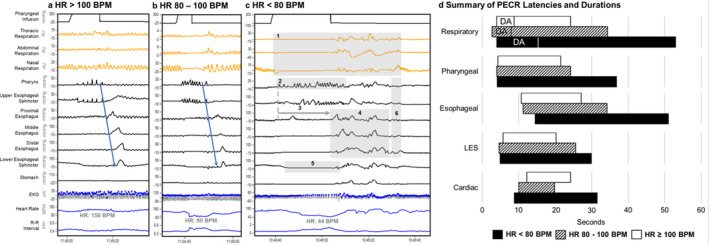
Pharyngeal infusion induced pharyngo‐esophageal cardio‐respiratory (PECR) reflexes during responses resulting in (a) no bradycardia (HR > 100 BPM), (b) bradycardia (HR 80– 100 BPM) and (c) severe bradycardia (HR < 80 BPM). Note the well‐coordinated, brisk, inter‐relationships between the airway‐digestive‐cardiac systems in (a) and (b) with the self‐regulated terminal swallow (blue arrow) that depicts rapid restoration of cardio‐respiratory and digestive normalcy. In contrast, note the level of dysfunction during (c) severe bradycardia characterized by: (1) prolonged pharyngo‐glottal closure manifested as apnea beginning as central and becoming obstructive (thus called mixed apnea) in nature, (2) increased pharyngeal activity, (3) prolonged esophageal body inhibition, (4) presence of polymorphic esophageal body activity, (5) prolonged LES relaxation, with (6) absence of a terminal propagating swallow. In clinical situations like these where self‐regulation and terminal swallow are absent, interventions by personnel may be necessary to restore normalcy in symptomatic cases. (d) Timing, sec, of PECR responses during (a ‐ c) are shown. DA, deglutition apnea portion of respiratory change. Bars represent regional response duration with the bar start signifying response onset and bar stop signifying response offset. The time interval between pharyngeal stimulus onset (0 sec) andresponse onset represents response latency for each region. Note the distinct regional responses with differing heart rates (see Figure 1d key), specifically, prolonged responses and delayed esophageal body activity with HR < 80 BPM. We hypothesize that differential activation via parasympathetic excitatory (ACh), inhibitory (NO/VIP) or sympathetic (adrenergic) systems may be contributory to this pharyngo‐esophageal‐cardio‐respiratory regulation. This may be at central neurological or peripheral neurological or both areas, which ultimately influences muscular activity

From the 287 pharyngeal infusions analyzed, responses resulting in HR < 80 BPM (*N* = 18) were present in 14 subjects (12 from recurrent bradycardia and two from control groups), HR 80–100 BPM (*N* = 24) were present in 18 subjects (14 from recurrent bradycardia and four from control groups), and HR > 100 (*N* = 245) were present in all 40 subjects. Comparison of PECR characteristics resulting in bradycardic rhythms are shown in Table 2. Additionally, (a) during severe bradycardia (HR < 80) the odds of a terminal peristaltic reflex being present is less likely versus HR 80–100 with OR (95% CI) of 0.2 (0.0–0.9), *p* = .03, and versus HR > 100 with OR (95% CI) of 0.2 (0.0–0.6), *p* < .01; (b) symptom occurrence (movement/arching or coughing) is more likely in HR < 80 versus HR > 100 with OR (95% CI) of 6.9 (1.1–44.6), *p* = .04; and (c) HR drop occurring before esophageal contractile activity is more likely with HR < 80 versus HR > 100 with OR (95% CI) of 5.9 (1.0–32.7), *p* = .04. None of the infants required severe intervention during bradycardic rhythms. The above results are summarized in Figure 1. Volumetric effect on HR was analyzed: For each 0.1 ml stimulus volume increase, HR drops by 63 ± 10 BPM (*p* < .01).

**TABLE 2:** Comparison of adaptive pharyngeal swallowing reflex characteristics stratified by bradycardic rhythm severity (compared to normal rates, HR > 100 BPM)

Characteristic	HR < 80 BPM (*N* = 18 stimuli)	HR 80–100 BPM (*N* = 24 stimuli)	HR > 100 BPM (*N* = 245 stimuli)	*p*‐value
Stimulus duration, s	9.1 ± 0.9	9.5 ± 0.8	8.3 ± 0.4	.42
Stimulus flow, ml/s	0.04 ± 0.01	0.05 ± 0.01	0.05 ± 0.00	.63
Pharyngeal phase				
Recruitment, # peaks/stimulus	9.3 ± 0.8*	7.3 ± 0.7	6.1 ± 0.4	<.01
Response latency, s	4.1 ± 0.6	4.0 ± 0.6	4.3 ± 0.4	.87
Response duration, s	32.7 ± 5.6*	20.2 ± 4.9	17.2 ± 2.0	.07
Frequency, Hz	0.6 ± 0.1	0.7 ± 0.1	0.6 ± 0.0	.67
Peak–peak variability, s	24.5 ± 3.9	18.6 ± 3.4	16.5 ± 1.6	.22
Stability, *SD*/ $\overset{-}{X}$	0.8 ± 0.1	0.7 ± 0.1	0.7 ± 0.0	.59
Esophageal phase				
Response latency, s	14.5 ± 1.6	11.2 ± 1.5	10.7 ± 0.7	.14
Inhibition, s	11.7 ± 1.4*	8.8 ± 1.3	7.3 ± 0.6	.01
Response duration, s	36.5 ± 6.0*	23.0 ± 5.5	16.4 ± 2.0	.01
LES				
Basal tone, mmHg	17.1 ± 2.3	17.8 ± 2.1	17.7 ± 1.3	.95
LESR response latency, s	4.9 ± 0.8	4.6 ± 0.7	5.7 ± 0.5	.20
LESR onset to nadir, s	1.8 ± 0.3	2.3 ± 0.3	2.0 ± 0.1	.62
LESR nadir duration, s	25.0 ± 2.7*	21.0 ± 2.3*	14.5 ± 1.1	<.01
LESR nadir pressure, mmHg	0.5 ± 1.1	0.2 ± 0.9	−0.1 ± 0.6	.87
Cardiac				
Basal HR, bpm	149 ± 2.5	151 ± 2.4	150 ± 2.0	.62
HR response latency, s	8.8 ± 1.4	10.0 ± 1.3	12.2 ± 1.0	.07
HR response duration, s	22.7 ± 3.6*,^†^	9.8 ± 3.1	12.0 ± 1.9	.03
HR decrease response magnitude, %	54.1 ± 1.6*,^†^	38.6 ± 1.4*	22.8 ± 1.0	<.01
Basal R–R interval, s	0.4 ± 0.01	0.4 ± 0.01	0.4 ± 0.00	.52
R–R response, s	0.9 ± 0.02*,^†^	0.6 ± 0.02*	0.4 ± 0.01	<.01
Respiratory				
Rhythm change latency, s	4.0 ± 0.7	2.7 ± 0.6	4.0 ± 0.3	.20
Rhythm change duration, s	49.1 ± 6.2*	31.6 ± 5.3	20.2 ± 2.0	<.01
Deglutition apnea duration, s	11.4 ± 1.5*,^†^	5.2 ± 1.3	4.7 ± 0.5	<.01

Note

Data presented as mean ± *SE* or as stated.

Note that during severe bradycardia (HR < 80 BPM): pharyngeal activity is greater (increased recruitment and duration), esophageal inhibition and response duration are prolonged, LES relaxation duration is prolonged indicating increased inhibition at the LES, duration of cardiac rhythm response is prolonged, and respiratory rhythm changes are prolonged. All these modulations suggest that central pattern generators (rhythm generators) for respiratory, cardiac and pharyngo‐esophageal regulation may have overlapping cross‐system neural interaction that is essential to preserve the autonomic balance in preterm infants.

Abbreviations: *SD*, standard deviation; $\bar{X}$, mean; LES, lower esophageal sphincter; LESR, lower esophageal sphincter relaxation; HR, heart rate; bpm, beats per minute.

* *p* < .05 versus stimuli resulting in HR > 100 BPM,

^†^ *p* < .05 versus stimuli resulting in HR between 80 and 100 BPM.

## 4 DISCUSSION

### 4.1 Salient findings

Difficulties with eating coupled with cardiorespiratory spells delay the acquisition of feeding milestones in convalescing neonates, and the mechanisms remain unclear. The aims of the current study were to examine and compare pharyngeal–esophageal–cardiorespiratory response characteristics (a) of infants with clinical history of recurrent bradycardia versus control; and (b) pharyngeal stimulation responses that actually resulted in bradycardia versus responses not resulting in bradycardia in infants convalescing in the neonatal ICU during transition to successful full oral feedings. The salient findings of the current study are as follows: (a) In regard to *clinical description of recurrent bradycardia events history*: (1) self‐recovery or mild intervention involving gentle stimulation by providers resolved 60% of bradycardic events, with the remaining 40% requiring moderate to severe intervention, and b) 44% events were feeding related, 3% lower gut related, and 53% occurred spontaneously. (2) *In infants with recurrent bradycardia versus control:* Minimal PECR differences are noted. (3)* In responses resulting in bradycardia:* (i) PECR responses are characterized by increased pharyngeal activity, increased esophageal dysmotility as evidenced by prolonged inhibition of motor activity and LES relaxation, prolonged cardiac response and respiratory change durations, and increased frequency of respiratory changes and symptoms. Additionally, PECR responses become magnified with increasing severity of HR decrease.

### 4.2 Physiological explanation of findings

In a prior study of 48 preterm NICU infants that achieved full oral feeds (suggestive of adequate functional maturation with aerodigestive coordinating skills), the prevalence of HR decrease (defined as 10% change from baseline) with pharyngeal stimulation was 32% at 39 weeks PMA (Hasenstab et al., 2019). Among the HR decrease responses: 13% resulted in HR < 100 BPM and 5% resulted in HR < 80 BPM (Hasenstab et al., 2019). HR decrease was, however, related to extreme preterm birth, repetitive swallowing, increased respiratory rhythm disturbance, and decreased esophageal propagation rates (Hasenstab et al., 2019). Those observations suggest the involvement of a possible downstream esophageal response pathway associated with cardiorespiratory events that is, apnea and bradycardia, that may contribute to worsening clinical presentation in convalescing preterm infants at full‐term maturational age. Physiological studies in infants and older subjects with bowel inflammation have described decreased HR variability in functional and inflammatory gastrointestinal disorders (Doheny et al., 2014; Polster et al., 2018). In rodents, a significant positive correlation between high frequency HR variability and gastric motility was detected (Meister, Jiang, Doheny, & Travagli, 2019). It was also found in newborn lambs that esophageal nociceptive stimulation by rapid balloon distention or HCl infusion has resulted in inhibitory cardiorespiratory reflexes combined with protective mechanisms which included laryngeal closure, swallowing, and coughing (Nault, Samson, Nadeau, Djeddi, & Praud, 2017). These cardiorespiratory events were heightened in preterm lambs compared to full‐term lambs. Collectively, these studies (Doheny et al., 2014; Hasenstab et al., 2019; Meister et al., 2019; Nault et al., 2017; Polster et al., 2018) corroborate with our study findings of abnormalities of PECR response characteristics during pharyngeal stimulus‐induced bradycardia compared to those with no bradycardic response in human infants. It is possible that pharyngeal–esophageal stimulation simultaneously induces cardiorespiratory reflexes (via brain stem vagal nuclei; Broussard & Altschuler, 2000), which may be more pronounced in preterm human infants even at full‐term maturation, thus signifying delays in specific eating milestones as well as delays with normalization of airway protection.

It is known that reciprocal relationships between pharyngeal and respiratory biorhythms exist at younger maturational age and aerodigestive safety metrics advance with PMA and growth. Researchers have studied the group of laryngeal chemoreflexes (LCR) in infants which may serve to protect against aspiration (Davies et al., 1985, 1989; Pickens et al., 1988), and may include startle, rapid swallowing, apnea, laryngeal constriction, hypertension, and bradycardia (Thach, 2001). The regulation of LCR, that is, onset and offset is modulated by vagal input as evidenced by concomitant pharyngoesophageal motility and cardiorespiratory rhythm changes (Broussard & Altschuler, 2000; Thach, 2001). Esophageal peristalsis occurs as a coordinated complex neuromuscular process, primarily driven by the interaction of the excitatory (cholinergic) and inhibitory (noncholinergic) parasympathetic pathways (Hellemans, Pelemans, & Vantrappen, 1981; Mashimo & Goyal, 2006; Esophageal, Peristalsis, & Motility, 2006).

Under normal circumstances, pharyngeal swallowing and esophageal peristalsis terminate respiratory dysregulation, and restores aerodigestive normalcy (Hasenstab et al., 2018, 2019; Jadcherla et al., 2016). However, during multiple pharyngeal swallowing events, esophageal peristalsis is inhibited until a final pharyngeal contraction occurs; this phenomenon is known as “deglutitive inhibition,” an inhibitory vagal response (Mashimo & Goyal, 2006). This final pharyngeal contraction induces sequential esophageal contractions (terminal swallow) mediated partly by the interaction of cholinergic (acetylcholine [ACh]) and noncholinergic (NO/VIP) pathways (Mittal, 2011). Lower esophageal sphincter usually relaxes after the onset of pharyngeal swallow, an inhibitory parasympathetic response via the nitrergic pathway (Goyal & Chaudhury, 2008). An alternative hypothesis is that when respiratory dysregulation stops and returns to normalcy, pharyngeal swallowing and esophageal peristalsis can result in clearance. However, such concepts can only be tested using bench research models. In such situations, the neural event which stopped the respiratory dysregulation may also have stimulated the peristalsis, or the cause of the peristalsis was the stoppage of dysregulation. Further studies are needed to test these hypotheses.

**FIGURE 2:**
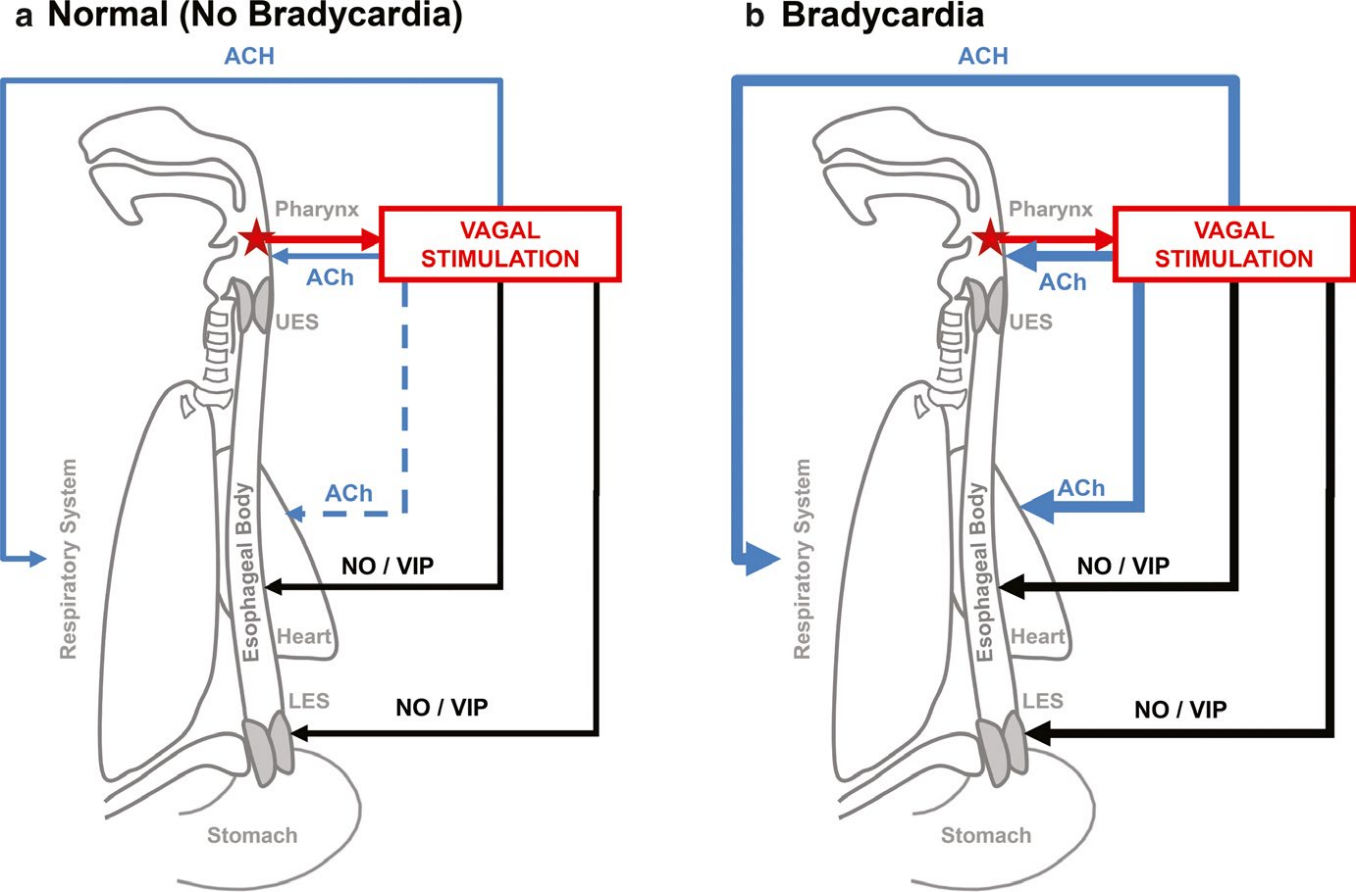
Hypothesis‐generating concepts for cardiac rhythm‐modifying circuitry. Blue line represents parasympathetic cholinergic pathways depicting acetylcholine (ACh) as the primary driver and black line represents parasympathetic non‐cholinergic pathways depicting nitric oxide or vasoactive intestinal peptide (NO/VIP) as the primary driver to the specified region. Weighted lines represent increased activation of that specific pathway. (a) In responses without bradycardia: pharyngeal stimulation (red arrow) activates vagal excitatory (ACh) and inhibitory (NO/VIP) pathways resulting in normal pharyngeal‐esophageal peristalsis and LES relaxation, in addition to respiratory autoregulation. The dashed blue arrow represents minimal or no effect on heart rate rhythm. (b) In responses with bradycardia: In contrast to (a), activation of pathways are increased. Also recall Figure 1c wherein pharyngeal stimulation was associated with apnea and bradycardia events along with abnormalities in PECR responses. Apnea and bradycardia causing mechanisms may be due to pharyngeal stimulus induced parasympathetic effects (ACh) via respiratory inhibition and cardiac vagal stimulation respectively. Concurrent abnormalities with pharyngeal contractility, delayed peristalsis, and prolonged LES relaxation may be due to increased inhibition (NO/VIP)

In the cardiovascular system, parasympathetic innervation of the heart is mediated by the vagus nerve via ACh; especially at the level of sinoatrial and atrioventricular nodes (Thompson et al., 1998). Its stimulation leads to negative chronotropic response “bradycardia.” During our study, infants in whom pharyngeal stimulation resulted in severe bradycardia, also exhibited pharyngoesophageal dysfunction and delays in restoring cardiorespiratory balance; such responses were directly correlated with severity of bradycardia. Apnea or altered breathing pattern was the initial step of a sequence of events; followed almost immediately by an abnormally prolonged pharyngeal phase, characterized by multiple and disorganized pharyngeal swallows. Decreased frequency of terminal swallows, delayed esophageal body response, and prolonged LES relaxation were also observed. We speculate that severe bradycardia is more likely to occur during the esophageal inhibition period. Remarkably, those infants with severe bradycardic response not only had longer esophageal inhibition, but also prolonged: polymorphic esophageal body activity, respiratory rhythm disturbance and bradycardia. We suggest that the constellation of clinical and manometry findings described, may be mediated by an exaggerated and disproportionate parasympathetic (vagal) response at pharyngeal‐esophageal and cardiorespiratory rhythms evoked upon pharyngeal stimulation (Figure 2).

### 4.3 Clinical and applied physiological implications

The combination of apnea and bradycardic spells with feeding difficulties are common clinical scenarios that often delay discharge of premature infants from NICUs resulting in an enormous burden to health care system and distress for parents. Given the concerns for increased risk of bradycardic events during feeds, clinicians are usually very cautious with initiation and progression of oral feeds in infants with history of recurrent bradycardia. However, it is unknown whether oral feeds worsen bradycardic events in premature infants with history of bradycardia.

We demonstrated that, following pharyngeal stimulation, infants with and without history of recurrent bradycardia have similar overall PECR response characteristics (Table 1), suggesting that pharyngeal stimulation per se does not always result in pharyngoesophageal dysfunction or increase risk of cardiovascular events regardless of the history of recurrent bradycardia. Therefore, we suggest that a history of recurrent apnea‐bradycardia alone should not be used by clinicians solely as a guide for initiation or progression of oral feeds.

Our results support the presence of an overlapping cross‐system neural interaction that is essential to preserve the autonomic balance in preterm infants. A summary of cardiorespiratory and motility timing relationships, during PECR responses resulting in bradycardic rhythms is shown (Figure 1d). Based on our findings, we postulate that prolonged deglutitive inhibition may potentially worsen infant's cardiorespiratory status with resulting increase in duration and severity of apnea and associated bradycardia. Thus, we consider that oral stimulation in infants experiencing cardiorespiratory dysregulations may be a key intervention in the restoration of cardiorespiratory normalcy by normalizing terminal swallows as the final event of stimulation.

Absent, decreased, or irregular breathing is one of the clinical findings used for diagnosis of brief resolved unexplained event (BRUE), a common condition responsible for multiple emergency room visits and hospital admissions in infants. It has also been described that feeding difficulties could potentially lead to BRUEs (Tieder et al., 2016). BRUE practice guidelines by the American Academy of Pediatrics provide management recommendations for low risk infants; however, there are no standardized recommendations for the high risk group patients like preterm infants born before 32 weeks of gestation, or patients with multiple BRUEs, who may be more likely to have adverse outcomes such as sudden infant death syndrome (Tieder et al., 2016). Our manometry findings provide a better understanding of the sequence of events potentially occurring during BRUEs in preterm born infants with feeding/swallowing difficulties and help identifying possible areas of interventions to acutely manage the cardiorespiratory event as well as to prevent their further occurrence.

Therefore, our study provides impetus for reverse translational studies aimed toward discovery of novel basic science mechanisms that may lead to potential drug development targeted toward cardiorespiratory homeostasis. Caffeine or theophylline is one such group of agents, although their use is not prolonged beyond preterm period (Abu‐Shaweesh & Martin, 2017). Prokinetics to enhance pharyngeal‐esophageal motility that are safe for neonatal use need further development.

### 4.4 Limitations and future directions

Physiological studies in human infant can have limitations as cellular level mechanisms cannot be answered, but rather will provide guidance toward translational studies. Owing to the unique histological structure (skeletal and smooth muscle) and extremely rich innervation (sympathetic and parasympathetic, intrinsic and extrinsic) in the esophagus, it is difficult to solely attribute all our findings of pharyngeal‐esophageal dysfunction to the vagus nerve alone, as cross‐system coordination can be implicated in our studies. We evaluated only those preterm infants close to full‐term PMA referred for feeding/swallowing assessment. While it is true that generalization is not yet possible, sound rationale can be proposed from our physiological observations.

Pulse oximetry data obtained during our study were not included; as tracings were not integrated with the PECR rhythm recordings. There were no safety concerns with our approaches as well as no side effects or consequences of cautious stimulation protocol with bolus volumes ≤ 0.5 ml. However, larger volumes may predict a larger difference in those with recurrent bradycardias. Further cautious protocols are needed which can be of diagnostic utility.

Strategies examining methods to stimulate eating and swallowing are needed in infants with a history of recurrent apnea‐bradycardia and feeding difficulties in order to restore timely acquisition of feeding milestones. Such prospective studies must also evaluate methods that have a potential in aborting cardiorespiratory perturbations.

## 5 CONCLUSIONS

The similarities in PECR characteristics in those with and without recurrent bradycardia suggest adaptational capability with vagal regulation of pharyngeal‐esophageal and cardiorespiratory rhythms. The mechanisms of severe bradycardia spells are related to abnormal prolongation of vagal inhibitory effects on cardiorespiratory rhythms in conjunction with prolonged esophageal inhibition and delays with terminal propagating peristaltic reflexes. Limiting cardiac vagal response in those infants, could lead to less severe drop in HRs, improved pharyngeal swallow and more efficient esophageal clearance, all contributing toward faster achievement of full oral feeds and cardiorespiratory stability.

## CONFLICT OF INTEREST

Guarantor of the article: Sudarshan R. Jadcherla; Any potential competing interests: None.

## AUTHOR CONTRIBUTIONS

The following specific contributions from individual authors are as follows: KAH and SRJ were involved in concepts and experimental study design, performed manometry studies, acquired data, performed data analysis, and developed the first manuscript draft. KAH, JBS, and VP performed literature review and developed tables and figures. VP performed statistical modeling design, and analysis. KAH, JBS, VP, and SRJ validated data and results. KAH, JBS, VP, IML, RS, and SRJ critically reviewed and interpreted data, involved in manuscript writing, editing, and approval of final version. SRJ, IML, and RS secured funding, and were co‐investigators who provided basic and clinical science concepts.
